# Soluble urokinase plasminogen activator receptor promotes endoplasmic reticulum stress and apoptosis susceptibility through RAGE in sepsis acute kidney injury

**DOI:** 10.1186/s10020-025-01352-w

**Published:** 2025-09-26

**Authors:** Bingqing Wang, Jiabei Wang, Chen Qi, Chao Gao, Yue Wang, Yujie Zan, Yuwei Tan, Zhenying Wu, Jun Jiang, Jinmeng Suo, Jing Zhang, Zhiyong Peng

**Affiliations:** 1 Department of Critical Care Medicine, Zhongnan Hospital of Wuhan University, 169 Donghu Road, Hubei, 430071 Wuhan China; 2 Department of Neurology, Renmin Hospital of Wuhan University, 99 Zhangzhidong Road, Hubei, 430060 Wuhan China; 3 Center of Structural Heart Disease, Zhongnan Hospital of Wuhan University, 169 Donghu Road, Hubei, 430071 Wuhan China; 4 School of Remote Sensing and Information Engineering, Wuhan University, 129 Luoyu Road, Hubei, 430070 Wuhan China

**Keywords:** Acute kidney injury, Soluble urokinase plasminogen activator receptor, Endoplasmic reticulum stress, Apoptosis

## Abstract

**Background:**

Acute kidney injury (AKI) is a common complication among critically ill patients, associated with an increased risk of adverse outcomes. There is an urgent need for novel biomarkers to assist in the early detection and management of AKI. Soluble urokinase plasminogen activator receptor (suPAR) is an inflammation-related, immune-derived molecule implicated in the pathogenesis of several diseases, including kidney diseases.

**Methods:**

We characterized the ability of serum suPAR levels to diagnose AKI in 124 patients admitted to the intensive care unit (ICU). Additionally, in vivo and in vitro experiments were performed to explore the underlying mechanisms between suPAR and the development of AKI. We stimulated HK-2 cells with suPAR to investigate its effects on HK-2 cells. Additionally, Methods such as receptor inhibitors, protein docking, and co—immunoprecipitation were used to study how suPAR acts on HK-2 cells. We further explored whether the uPAR monoclonal antibody could alleviate acute kidney injury in septic mice.

**Results:**

We found that serum suPAR levels were significantly elevated in patients with AKI. In addition, total suPAR/uPAR was elevated in the renal cortex of AKI mice, and serum suPAR levels were also increased. In vitro cell experiments demonstrated that suPAR stimulation promoted endoplasmic reticulum stress (ER stress) and the expression of apoptosis—related proteins in HK-2 cells and increased intracellular reactive oxygen species (ROS). Consistently, mice injected intraperitoneally with recombinant suPAR also exhibited elevated ER stress in the renal cortex. Furthermore, we discovered that suPAR was immunoprecipitated with the receptor of advanced glycation end products (RAGE), and recombinant suPAR labeled with FITC was fluorescently colocalized with RAGE on HK-2 cells, indicating that RAGE was involved in the signal transduction of suPAR. Additionally, protein docking results showed that suPAR can form a protein–protein complex with RAGE through hydrogen bonds. Pretreatment with uPAR monoclonal antibody alleviated kidney injury in septic mice and reduced the levels of ROS, apoptosis, and endoplasmic reticulum stress in the kidneys of septic AKI mice.

**Conclusions:**

Our study demonstrates that high levels of suPAR are positively correlated with the occurrence of AKI. suPAR promotes endoplasmic reticulum stress and increases susceptibility to apoptosis in renal tubular epithelial cells. RAGE on the cell membrane can bind to suPAR, participating in the activation of suPAR-mediated endoplasmic reticulum stress pathways and the expression of apoptosis-related proteins. Pretreatment with uPAR monoclonal antibody alleviates acute kidney injury in septic mice.

**Supplementary Information:**

The online version contains supplementary material available at 10.1186/s10020-025-01352-w.

## Background

Acute kidney injury (AKI) is a common complication among critically ill patients, and its incidence is rising globally (Lameire et al. 2013). AKI is associated with an increased long-term risk of adverse outcomes, such as death, the incidence of chronic kidney disease (CKD), and higher utilization of health resources (Bellomo et al. 2012; Rewa and Bagshaw 2014). Currently, the diagnosis and staging of AKI still rely on serum creatinine (SCr) and urine volume, which have a certain lag (Rewa and Bagshaw 2014; Nusshag et al. 2017), thus limiting their clinical value in early diagnosis and disease related decision making.

In the newly proposed definition of AKI, it is recommended to combine stress, functional, and damage biomarkers for the diagnosis and management of AKI (Ostermann et al. 2020). Stress biomarkers like tissue me talloproteinase-2 (TIMP-2) and insulin-like growth factor binding protein 7 (IGFBP7), and damage biomarkers like kidney injury molecule 1 (KIM-1) and neutrophil gelatinase–associated lipocalin (NGAL) are all widely noticed (Ostermann et al. 2020; Strauß et al. 2024). These biomarkers have been found to not only serve as predictive indicators of AKI but also participate in the occurrence and development of AKI (Wen and Parikh 2021). Given that oxidative stress and inflammation, which involve multiple subtypes of immune cells, are vital components of the pathogenesis of AKI (Tran et al. 2016; Rabb et al. 2016), the inflammation—related, immune—derived molecule soluble urokinase plasminogen activator receptor (suPAR) has been under exploration as a novel potential biomarker for AKI, and its role in AKI is currently being investigated as well. (Hayek et al. 2020; Nusshag et al. 2023; Azam et al. 2020).

urokinase plasminogen activator receptor (uPAR) is a highly glycosylated, lipid anchored receptor on the surface of various cell types (Thunø et al. 2009), including immune cells and vascular endothelial cells, and it is involved in cell adhesion, migration, invasion, and tissue remodeling (Blasi and Carmeliet 2002). Infection and other stimulants upregulate the expression of uPAR in immune cells and uPAR participate in the activation and migration of these cells (Mondino and Blasi 2004; Gussen et al. 2019; Montuori and Ragno 2009). Soluble urokinase plasminogen activator receptor (suPAR) is its soluble biologically active form, which is cleaved by GPI specific phospholipase C or cathepsin G (van Veen et al. 2017; Ploug et al. 1991). An increasing number of studies are exploring the potential of suPAR to serve as a biomarker of AKI or systemic inflammation and high blood levels of suPAR are closely related to AKI (Hayek et al. 2020; Nusshag et al. 2023; Azam et al. 2020; Nusshag et al. 2019; Backes et al. 2012; Rasmussen et al. 2021). Currently, research have found various functions of suPAR, including the inhibition of neutrophil exocytosis, binding to urokinase plasminogen activator (uPA) and vitronectin, stimulation of angiogenesis through endothelial sprouting and tube formation, promotion of chemotaxis, and interaction with β3 integrin leading to glomerular podocyte injury (Rasmussen et al. 2021). Moreover, it was found that suPAR may modulate cellular bioenergetics and increase oxidative stress, sensitizing kidney proximal tubules to injury (Hayek et al. 2020). Additionally, suPAR has been proved to bind to integrin β6 on tubular cells, leading to Rac1 activation followed by an onset of CD44/Smad3 signaling, culminating in interstitial fibrosis (Han et al. 2019). Besides, suPAR can exert its functions through membrane receptors such as Toll-like receptor 4 (TLR4), receptor of advanced glycation end products (RAGE), and integrins (Kim and Dryer 2021; Huang et al. 2023). However, the pathophysiological role of high levels of serum suPAR in acute kidney injury is not yet fully understood.

In this study, we investigated the predictive value of suPAR for AKI as well as its underlying mechanisms involved in the development of AKI. We found that the serum suPAR level in patients is positively correlated with the occurrence of AKI and could assist in AKI prediction. In addition, our results indicate that suPAR promotes endoplasmic reticulum stress (ER stress) through the RAGE receptor, increasing the susceptibility of renal tubular epithelial cells to apoptosis. Pretreatment with uPAR monoclonal antibody can alleviate septic acute kidney injury in mice.

## Materials and Methods

### Research design and oversight

This was an observational study carried out in Zhongnan Hospital of Wuhan University. The study was approved by the Ethics Committee of Zhongnan Hospital of Wuhan University (approval number: #2,021,080). The experiments were conducted in the intensive care unit (ICU) from April 2021 to April 2022. The serum concentrations of suPAR at enrollment and the serum creatinine levels (SCr) at enrollment and 24, 48 h after enrollment were measured. The suPAR ELISA kit was provided by Elabscience (Cat No: E-EL-H2584c). Serum cystatin C (Cys-C) and β2-microglobulin (β2-MG) levels were quantitatively measured using latex-enhanced immunoturbidimetric assays at the Clinical Laboratory of Zhongnan Hospital of Wuhan University. The primary outcome measure was AKI or death within 7 days starting from enrollment. AKI was defined according to the 2012 KDIGO guidelines as follows (Khwaja 2012): 1. An absolute increase in the creatinine level of at least 0.3 mg per deciliter (30 μmol per liter) within 48 h. 2. A relative increase of at least 50% in the creatinine level within the previous 7 days. 3.Less than 0.5 ml/kg/h of urine volume for 6 h.

### Patient enrollment

Patients admitted to the ICU were screened according to the following inclusion criteria: 1) Aged above 18 years old; The exclusion criteria mainly encompassed the following situations: 1) patients in CKD5 stage or with a history of kidney transplantation; 2) pregnant women; 3) patients who died within 48 h after enrollment; 4) patients who refused to participate.

### Animal experiments

The 6-8w C57BL/6 mice were purchased from Vital River Laboratory Animal Technology Co. Ltd., Beijing, China. All mice were housed in sterile environments. They had free access to food and water, and were kept in standard cages with a 12—hour light—dark cycle. All animal experiments in this study were carried out in accordance with the protocol approved by the Animal Ethics Committee of the Animal Experimental Center of Wuhan University (WP20230557). All the mice were tissue—sampled and sacrificed under pentobarbital anesthesia. The septic AKI model was established by intraperitoneal injection of lipopolysaccharide (Ultrapure LPS, E. coli, InvivoGen) (dissolved in sterile saline) at a dose of 10 mg/kg. Cecal ligation and puncture (CLP) surgery was performed strictly as described in the literature (Rittirsch et al. 2009). A new cytokine storm—induced AKI model was attempted by intraperitoneal injection of TNF—α (10 μg/mouse, MedChemExpress, HY-P7090) and IFN—γ (20 μg/mouse, MedChemExpress, HY-P7071) (Karki et al. 2021). Blood samples and bilateral kidneys were collected at 24 h post-surgery, and at 6, 12, 24 and 48 h post-injection, respectively. The uPAR recombinant protein (20 μg/mouse, MedChemExpress, HY-P77276) was administered to mice via intraperitoneal injection. The uPAR monoclonal antibody (500 µg/kg, R&D system, MAB531) was injected intraperitoneally 5 min before AKI modeling. The mouse suPAR ELISA kit (ED-21788) was purchased from LunChangShuoBiotech (Xiamen, China).

### Cell culture

HK-2 cells were purchased from and identified by the Cell Bank of the Chinese Academy of Sciences (GNHu47). The cells were cultured in Dulbecco's Modified Eagle Medium/Nutrient Mixture F—12 (DMEM/F12, HyClone) supplemented with 10% fetal bovine serum (FBS), and the HK-2 cells were incubated at 37 ◦C and 5% CO2. 10 ng/ml recombinant suPAR (MedChemExpress, HY-P72433) was used to stimulate HK-2 cells for 6 h, 12 h and 24 h. FPS-ZM1 (100 nM), TLR4-IN-C34 (10 μM), and CWHM-12 (10 nM) were used to selectively inhibit RAGE, TLR4, and integrins, respectively (MedChemExpress, HY-19370, HY-107575, HY-18644).

### ROS measurement

1 × 10^5^ HK—2 cells were inoculated into a six—well plate and then stimulated with 10 ng/ml suPAR for 6 h, 12 h, and 24 h respectively. After being washed with PBS, HK—2 cells were incubated with DCFH—DA (Beyotime, S70033S), which was diluted with serum—free medium at a ratio of 1:8000, for 20 min at 37 °C. Subsequently, the ROS level in each sample was detected using flow cytometry (Beckman) and the data were analyzed with FlowJo software (FlowJo_v10.10.0; Becton, Dickinson and Company). Approximately 1 × 10^4^ cells in each sample were analyzed.

### Serum biomarkers of kidney injury

SCr and blood urea nitrogen (BUN) levels were measured using commercial kit reagents (Jiancheng Bioengineering Institute). The absorbance at 546 nm was set to measure the level of SCr and BUN via a multimode plate reader (Enspire, PerkinElmer).

### Histology and immunohistochemistry

Mouse kidney samples were fixed in 4% paraformaldehyde and then embedded in 10% paraffin. The paraffin—embedded renal blocks were cut into 3—μm—thick sections for periodic acid-Schiff staining (PAS) and immunohistochemistry. For immunohistochemical staining, the sections were incubated overnight with uPAR antibodies (abcam, ab307895, 1:100) at 4 °C. Images of both PAS and immunohistochemical staining were captured and analyzed using CaseViewer 2.4 software.

### Tubular injury score

Tubular injury score was evaluated in renal tissue stained with PAS. Renal tubular damage was categorized into six grades according to the criteria of brush border loss, tubular dilation, cast formation, tubular necrosis. Specifically, ten high-power fields were randomly selected, including five from the renal cortex and five from the cortico-medullary junction. Each field was scored on a 0–5 scale(0: normal; 1: mild injury, involvement of 0%–10%; 2: moderate injury, involvement of 11%–25%; 3: severe injury, involvement of 26%–49%; 4: high severe injury, involvement of 50%–75%; 5: extensive injury, involvement of > 75%). All evaluations were conducted by two investigators who were blinded to the experimental conditions to ensure objectivity.

### LTL staining score

Proximal tubular injury was evaluated using a semi-quantitative scoring system based on Lotus tetragonolobus lectin (LTL) staining patterns. The scoring criteria were defined as follows: Grade 0 (Normal) exhibited continuous and intense linear LTL staining along the apical membrane; Grade 1 (Mild) showed focal fragmentation or discontinuity affecting less than 50% of the tubule length; Grade 2 (Moderate) demonstrated disrupted staining involving more than 50% of the tubule length; while Grade 3 (Severe) was characterized by complete absence of LTL signal or presence of necrotic tubules. For each experimental group, nine representative images were analyzed by two independent investigators who were blinded to the experimental conditions.

### Immunofluorescence

The kidney tissue sections were dewaxed with xylene and then hydrated with ethanol. Subsequently, they were immersed in antigen repair buffer, boiled for 3 min, and allowed to cool naturally. After that, the kidney tissue slices or cell climbing slices were blocked and then incubated with the primary antibodies, and the secondary antibodies in sequence. Next, they were washed with 1 × phosphate—buffered solution. Additionally, TUNEL and ROS staining were conducted according to the protocol of commercial kits (Beyotime, C1088, S0033). Immunofluorescence images were captured using fluorescence microscopy and analyzed with ImageJ software.

### Western blot

Samples containing radioimmunoprecipitation assay (RIPA) buffer were incubated for 15 min and then centrifuged. Subsequently, the supernatant was collected. After determining the protein concentration of the samples using the bicinchoninic acid method, the samples were mixed with sodium dodecyl sulfate loading buffer and boiled at 95 °C for 10 min. Equal amounts of protein were separated by sodium dodecyl sulfate–polyacrylamide gel electrophoresis and transferred to a polyvinylidene fluoride membrane. After being blocked with 5% bovine serum albumin solution, the membrane was incubated with the following primary antibodies: uPAR (ab307895, abcam, 1:1000), P-PERK (29,546–1-AP, ProteinTech, 1:500), PERK (TN25331, Abmart, 1:1000), P-EIF2α(3398, CST, 1:1000), ATF4 (T55873S,Abmart, 1:1000), CHOP (T56694S, Abmart, 1:1000), Nrf2 (16,396–1-AP, ProteinTech,1:2000), HO-1 (27,282–1-AP, ProteinTech, 1:1000), P-p65 (3033, CST, 1:1000), p65 (8242,CST, 1:1000), BAX (50,599–2-Ig,ProteinTech,1:2000), BCL-2 (T40056F, Abmart, 1:1000), Actin (66,009–1-Ig, ProteinTech, 1:20,000), RAGE (16,346–1-AP, ProteinTech, 1:2000), His (66,005–1-Ig, ProteinTech, 1:5000), Caspase-3 (14,220, CST, 1:1000), cl-Caspase3 (9661, CST, 1:1000). After 4 °C overnight, the strips of membrane were washed with 1 × TBST solution and then incubated with the corresponding secondary antibodies (1:5000 dilution) at room temperature for 1 h. The bands of proteins were developed by an electrochemiluminescence imaging system (Tanon—5200, China).

### Analysis of relative gene expression using quantitative real-time PCR (RT-qPCR)

Total RNA was isolated from tissues or cells with the MolPure TRIeasy Plus Total RNA Kit (YEASEN, 19211ES60). Total RNA was reverse—transcribed into cDNA following the instructions of a qPCR RT Kit (YEASEN, 11141ES60). The cDNA was then subjected to RT—qPCR using the Taq Pro Universal SYBR qPCR Master Mix (Vazyme, Q712-02). Quantification was achieved by comparing the Ct values of each sample, which were normalized to β—actin. The sequences of the PCR primers used are presented in Supplementary Table 2.

### Immunoprecipitation

The magnetic beads coated with protein A + G (ThermoFisher,88,804) were placed on a rotary mixer together with the target proteins or IgG antibody and incubated overnight at 4 degrees Celsius. Subsequently, the lysate containing the protein sample was added to the complex and allowed to incubate overnight at 4 degrees Celsius as well. Proteins that were capable of interacting with the target proteins were pulled down simultaneously. After that, the complex was washed and then incubated with 1 × sodium dodecyl sulfate—polyacrylamide gel electrophoresis (SDS—PAGE) loading buffer at 100 degrees Celsius for 10 min. Subsequently, the supernatant was collected. The type and expression of the pulled—down proteins were identified through western blotting.

### Transmission electron microscope (TEM)

Kidney tissues or HK-2 cells that had been fixed with 2.2% glutaraldehyde were scraped into tubes and then stored at 4 °C. The samples were subsequently washed with an osmium tetroxide solution and re-fixed. After that, they were prepared into nano-sized sections, which were stained with uranium acetate and lead citrate respectively. Following the staining, the sections were washed with distilled water and then observed under an electron microscope (JEM-1400 PLUS, Japan).

### Protein docking

The protein structures of RAGE (PDB ID:6XQ1) and UPAR (PDB ID: 2FD6) were downloaded from the PDB database, and the interaction pattern of RAGE and uPAR was studied using the Hdock (Yan et al. 2017). Pymol 2.3.0 is used to analyze interaction patterns for docking results.

### Statistical analysis

The results of our study were analyzed using SPSS 26.0 software and python 3.8. Continuous variables were presented as the mean (standard deviation, SD) for normally distributed data and as the median (along with the quartile range) for non-normally distributed data, respectively. Categorical variables were expressed as percentages. For continuous variables, the analysis of variance was employed when the data followed a normal distribution, while the Kruskal–Wallis test was used for non-normally distributed continuous variables. For categorical variables, the chi-square test was utilized. Logistic regression was applied to characterize the association between the suPAR levels and AKI. The robustness of the suPAR diagnostic cutoff (determined by Youden's index) was evaluated using leave-one-out cross-validation (LOOCV) implemented in Python 3.8. For two-group comparisons, unpaired t-test (parametric) or Mann–Whitney U test (nonparametric) was used. Multiple group comparisons employed One-way analysis of variance (ANOVA) along with the least squares difference (L.S.D) test (parametric) or Kruskal–Wallis with Dunn's test (nonparametric), with preference given to nonparametric tests for small sample sizes (n = 3). The threshold for statistical significance was set at P < 0.05.

## Results

### Diagnostic value of serum suPAR level for acute kidney injury

**Fig. 1:**
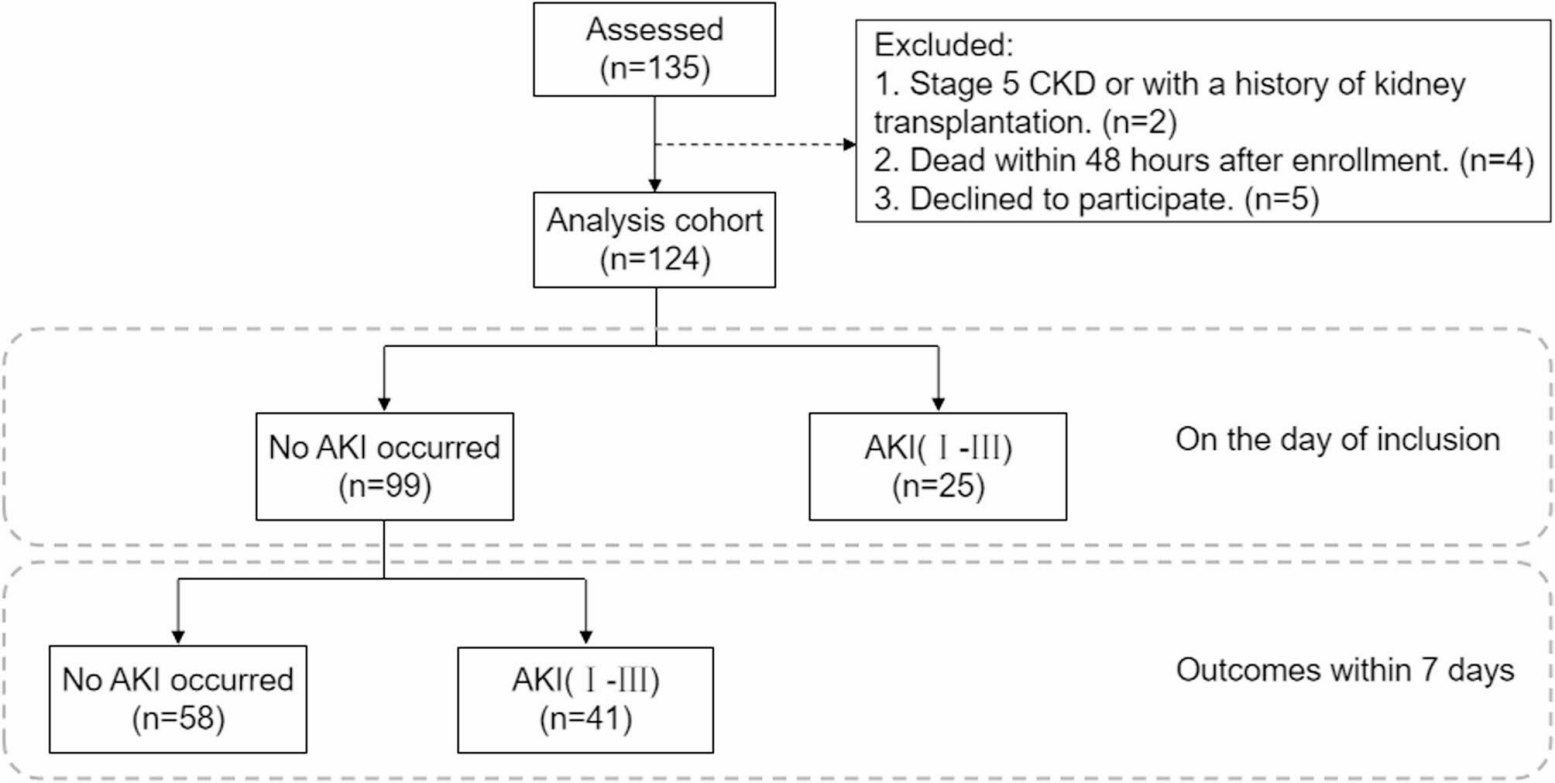
Flow chart of study design. AKI, acute kidney injury according to KDIGO criteria

Among the 135 patients admitted to the intensive care unit (ICU) under evaluation, 124 patients met the inclusion criteria. At enrollment, 25 patients presented with AKI. Among those patients without AKI at enrollment, 41 patients progressed to grade I—III AKI within seven days, while 58 patients remained free of AKI throughout the study period (Fig. 1). The baseline characteristics among the three groups were compared. It was found that, compared with patients without AKI, those with AKI were more likely to have sepsis and history of chronic liver disease at enrollment, as well as higher serum suPAR and creatinine levels. There were no differences in baseline demographics, other previous comorbidities, or other ICU admission details among the three groups (Table 1).

**Table 1:** Demographic and Clinical Characteristics of Enrolled Patients^§^

Characteristic	No Acute Kidney Injury(*N* = 58)	AKI occurred after enrollment(*N* = 41)	AKI had occurred at enrollment(*N* = 25)	*P* Value
Demographics				
Age — yr	61.0 ± 14.4	64.1 ± 15.5	62.3 ± 17.2	0.519
Male sex — no. (%)	37(63.8)	29(70.7)	15(60.0)	0.637
Body-mass index, kg/m2	22.3 ± 4.6	23.9 ± 4.5	22.3 ± 2.7	0.155
Comorbidities, n/*N* (%) ^ʉ^				
Chronic kidney disease	1/58(1.7)	3/40(7.5)	4/25(16.0)	0.051
Type 2 diabetes mellitus	9/58 (15.5)	5/40(12.5)	6/25 (24.0)	0.463
COPD*	6/58 (10.3)	2/40(5.0)	0/25 (0.0)	0.193
Heart failure	4/58 (6.9)	5/40(12.5)	2/25 (8.0)	0.623
Chronic liver disease	2/58 (3.4)	7/40(17.5)	1/25 (4.0)	0.031
Hypertension	16/47(34.0)	16/36(44.4)	12/25 (48.0)	0.444
Anaemia	7/58 (12.1)	6/40(15.0)	2/25 (8.0)	0.703
Primary diagnosis for ICU^#^ admission, n (%)				
Neurologic	9(15.5)	7(17.1)	4(16.0)	0.979
Respiratory	13(22.4)	8(19.5)	1(4.0)	0.123
Cardiovascular	6(10.3)	3(7.3)	3(12.0)	0.800
Trauma	14(24.1)	7(17.1)	3(12.0)	0.396
Gastrointestinal	4(6.9)	4(9.8)	0(0.0)	0.289
Shock	1(1.7)	5(12.2)	1(4.0)	0.078
Sepsis	6(10.3)	9(22.0)	11(44.0)	0.003
Relevant examinations at enrollment				
Median serum creatinine (IQR)—μmol/L	66.0(54.0–86.3)	84.0(71.0–107.0)	133.0(111.0–180.0)	< 0.001
Median suPAR^ǂ^ level (IQR)—ng/ml	3.6(2.6–4.4)	4.7(3.6–6.2)	6.1(4.6–7.5)	< 0.001

^**§**^Plus–minus values are means ± SD, IQR denotes interquartile range

^ʉ^ Comorbidities are presented as confirmed cases/available patients (percentage). Hypertension status was undocumented in 16 patients, among whom one lacked records for all comorbidities

^*^Chronic obstructive pulmonary disease (COPD)

^#^ Intensive care unit (ICU)

^ǂ^ Soluble urokinase plasminogen activator receptor(suPAR)

**Fig. 2:**
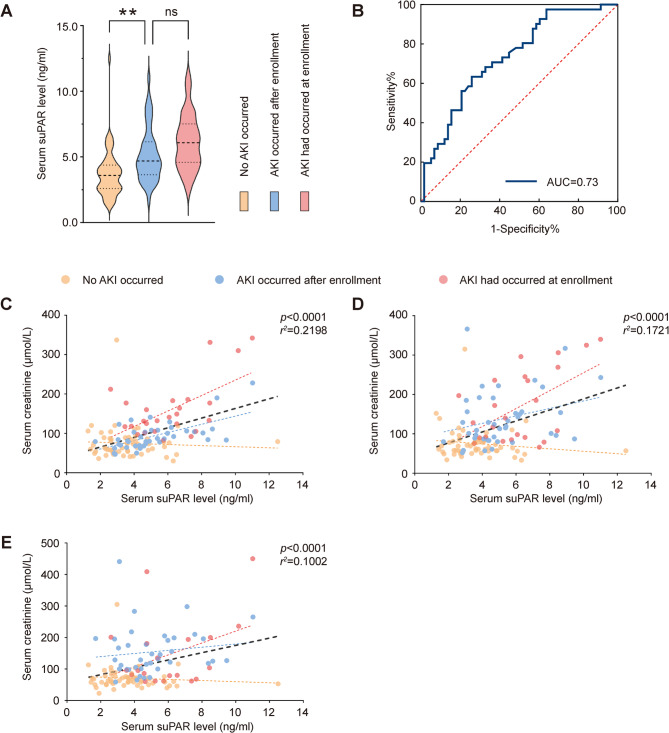
The association between suPAR and acute kidney injury among ICU patients. **A** Comparison of serum suPAR levels at enrollment among patients in the three groups, **P < 0.01. **B** After excluding the patients with pre-existing AKI at the time of enrollment, the ROC analysis was performed on the suPAR levels at enrollment, with the dichotomous variable being the occurrence of AKI. The area under the curve was 0.73. **C**-**E**. Correlations between the serum suPAR level at enrollment and the serum creatinine levels at the time of enrollment, 24 h after enrollment, and 48 h after enrollment, respectively

Serum suPAR levels of the three groups of patients on the day of enrollment are presented in Fig. 2A. Both patients who developed AKI within seven days after enrollment and patients with pre—existing AKI during enrollment have higher suPAR levels than those who did not develop AKI, and the differences are statistically significant. Meanwhile, the difference in serum suPAR levels between patients who developed AKI after enrollment and those who already had AKI at enrollment was not significant. To evaluate the diagnostic value of suPAR, after excluding the patients (n = 25) with AKI at the time of enrollment, we performed receiver operating characteristic (ROC) curve analysis and calculated the area under the curve to be 0.73 (Fig. 2B). Additionally, the optimal cut-off value of 4.28 ng/ml was determined by the maximum Youden's index from the ROC curve, with a sensitivity of 63.4% and a specificity of 74.1%. Stability of the cut-off value was confirmed by leave-one-out cross-validation (LOOCV) (mean cut-off: 4.33 ng/mL, 95% CI: 4.32–4.46). Using this threshold, patients with suPAR ≥ 4.28 ng/mL had 4.90-fold higher AKI risk (OR = 4.90, 95% CI: 2.02–11.87; χ^2^ p < 0.001). Furthermore, in a separate ICU validation cohort (n = 50, comprising 33 non-AKI and 17 AKI patients, Supplementary Table 1), We performed an ROC analysis comparing suPAR to two established kidney injury biomarkers: cystatin C (CysC) and β2-microglobulin (β2-MG).The analysis revealed comparable diagnostic performance between suPAR (AUC = 0.74) and CysC (AUC = 0.75), while β2-MG exhibited superior discriminative power (AUC = 0.85), as presented in Supplementary Fig. 1. A univariate and multivariate logistic regression analysis was conducted for suPAR, and the OR value was 1.52(95% Confidence Interval [CI], 1.18 to 1.97, p < 0.01). Serum suPAR levels at enrollment also correlated with SCr levels at enrollment as well as 24 h and 48 h after enrollment (*p* < 0.01, Fig. 2C, D and E). While the statistically significant association confirms suPAR's role in AKI discrimination, this modest effect size suggests limited utility as a standalone diagnostic biomarker.

### Total suPAR/uPAR increased in SA-AKI mouse kidney

**Fig. 3:**
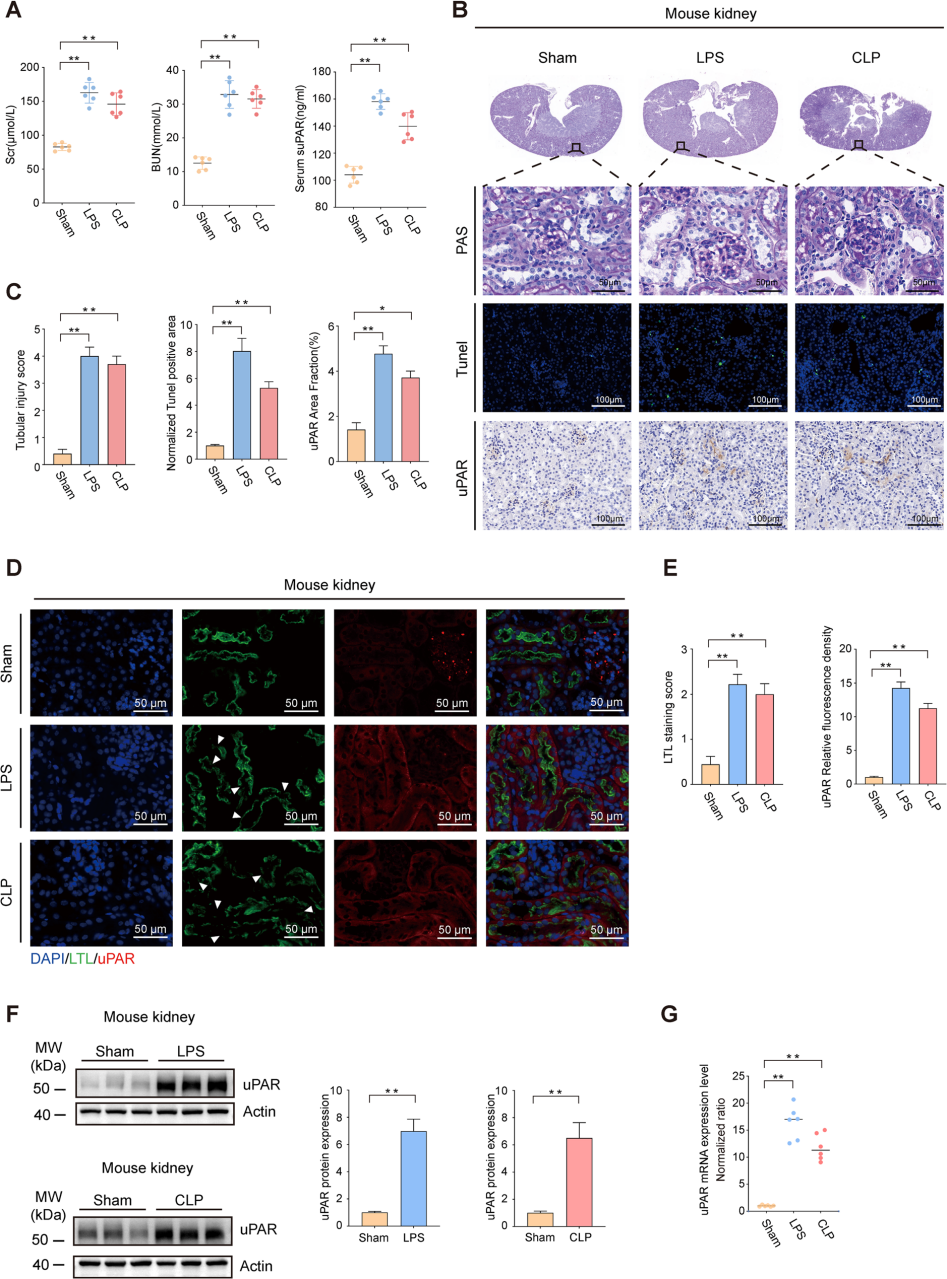
Increased suPAR/uPAR protein and mRNA levels in the kidneys of SA-AKI mice. **A** Serum levels of SCr, BUN, and suPAR in mice injected with saline (100 μL), LPS (10 mg/kg, 24 h), or subjected to CLP (24 h) (*n* = 6 mice/group). **B-C** Representative images of PAS staining, TUNEL staining, and uPAR immunohistochemical staining in kidney tissues across experimental groups, along with quantitative analyses (*n *= 3 mice/group; 3–10 fields/mouse). **D**-**E** uPAR immunofluorescence staining and quantitative assessment were performed in different groups of mice, with LTL staining used to visualize the brush border of renal tubular epithelial cells (White arrows: brush border discontinuity) (*n *= 3 mice/group; 3 fields/mouse). **F** Protein expression levels of suPAR/uPAR in kidney tissues from different groups (*n* = 3 mice/group; Mann–Whitney U test). **G** mRNA expression levels of uPAR in kidney tissues from different groups (*n* = 6 mice/group). Data were presented as mean ± SEM. Statistical significance was determined by one-way ANOVA or unpaired two-tailed Student's t-test unless noted, with *P* < 0.05 considered significant (*P < 0.05, **P < 0.01)

After confirming the elevation of serum suPAR level in AKI patients, in order to further investigate the pathophysiological mechanisms of how suPAR is implicated in AKI, we first explored the protein level of suPAR/uPAR in the kidneys of AKI mice. We established the mouse sepsis—associated acute kidney injury (SA-AKI) model by intraperitoneal injection of LPS (24 h) and CLP (24 h). The serum SCr and BUN levels of the mice treated with these two methods were much higher than those in the normal saline injection group (Fig. 3A). Meanwhile, the serum suPAR levels in two groups of SA-AKI mice were significantly higher than those in the control group, and the mice in the intraperitoneal injection of LPS group showed higher serum suPAR levels (Fig. 3A). PAS staining was performed to confirm the structure and morphology of the renal tubules. We observed edema, vacuoles, exfoliation of the brush border of the luminal surface in the dilated renal tubules. Renal tubular injury scores were also significantly higher in these two groups (Fig. 3B, C). After TUNEL staining, an increase in the degree of renal tubular cell apoptosis (more pronounced with LPS treatment) was also observed (Fig. 3B, C). Then, we detected the protein level of uPAR/uPAR in the mouse kidney by immunohistochemistry, immunofluorescence, and western blot. LTL staining was used to show the brush border of renal tubules. The results shown in Figs. 3B-E indicated that in healthy mice, uPAR/uPAR was mainly concentrated in the glomerulus, while in the two SA-AKI models, the total amount of suPAR/uPAR in the mouse renal tubules was also significantly increased. Moreover, the higher protein level of suPAR/uPAR in the renal tubules of the two SA-AKI groups corresponds to more prominent damage to the brush border (Fig. 3D, E). We also examined the protein lysate of the renal cortex in AKI mice and confirmed the elevation of suPAR/uPAR in injured kidneys (Only suPAR/uPAR at 50–65 kDa was detected in our study, which primarily represents full-length suPAR and uPAR) (Fig. 3F). Additionally, the mRNA expression levels of uPAR in the renal cortex of the two groups of SA-AKI mice were significantly increased (Fig. 3G). Furthermore, our LPS time-gradient model demonstrated that the total suPAR/uPAR level was detectably elevated at 6 h post-LPS injection, reached its peak at 12 h, and remained significantly elevated between 6–48 h (Supplementary Fig. 2G). At the same time, we attempted to explore acute kidney injury in the cytokine storm model by intraperitoneal injection of TNF—α and IFN—γ (for 6 h) (Karki et al. 2021). This model also effectively recapitulated AKI features in mice, including elevated serum SCr and BUN, exacerbated histological damage, increased cellular apoptosis. Notably, the upregulation of suPAR/uPAR levels in the renal cortex of mice was also detected in this cytokine storm-induced AKI model (Supplementary Fig. 2A-F).

### suPAR promotes apoptosis susceptibility in renal tubular epithelial cells through ER stress and oxidative stress

**Fig. 4:**
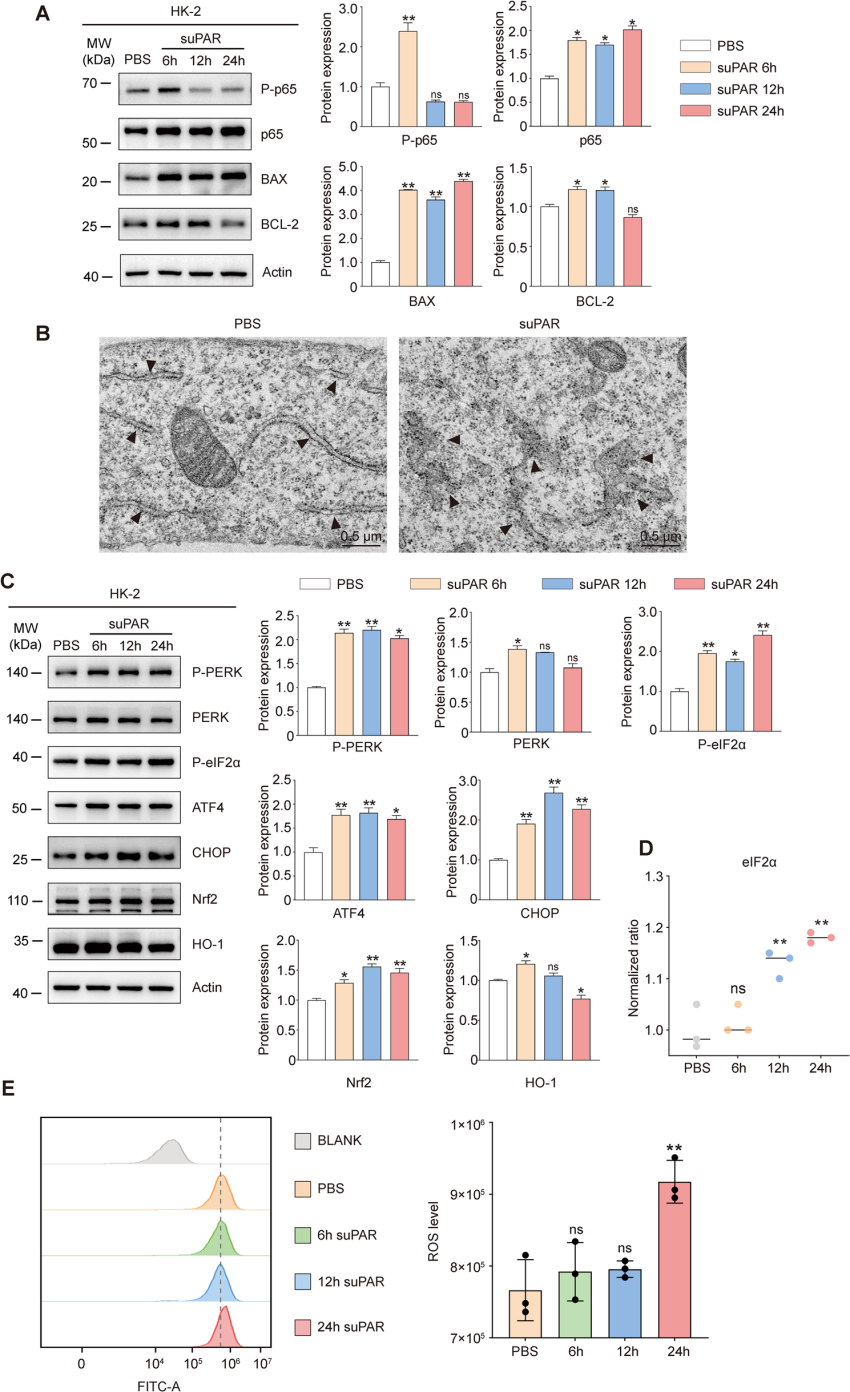
suPAR promotes apoptosis susceptibility in renal tubular epithelial cells through ER stress and oxidative stress. **A** Protein levels of P-p65, p65, BAX, and BCL-2 in HK-2 cells treated with suPAR (10 ng/mL) at different time points. **B** Representative images of transmission electron microscope for endoplasmic reticulum in tubular epithelial cells (The black arrow refers to the endoplasmic reticulum). Scale bar: 0.5 μm. **C** Protein levels of P-PERK, PERK, P-eIF2α, ATF4, CHOP, Nrf2, and HO-1 in HK-2 cells treated with suPAR (10 ng/mL) at different time points. **D** mRNA expression levels of eIF2α in HK-2 cells at different time points. **E** Intracellular ROS levels in HK-2 cells at different time points. Data were presented as mean ± SEM from three independent experiments (*n* = 3). Statistical significance was determined by Kruskal–Wallis test with Dunn's post hoc analysis, with *P* < 0.05 considered significant (*P < 0.05, **P < 0.01)

Given that there is a higher concentration of suPAR/uPAR in the damaged renal tubules, we tried to explore whether suPAR can directly act on renal tubular epithelial cells. After stimulating HK-2 cells with suPAR for different durations (6 h, 12 h, 24 h), we found that suPAR promoted the transient activation of Nuclear Factor Kappa-light-chain-enhancer of Activated B Cells (NF-κB). NF-κB is a classic inflammatory and stress factor and is involved in the regulation of cell death, proliferation, regeneration, glucose metabolism, and ROS handling (Guo et al. 2024). In addition, the protein levels of mitochondrial apoptosis—related proteins Bcl-2-Associated X Protein (BAX) and B-cell lymphoma/leukemia-2 (BCL-2) increased and decreased over time (Tsujimoto 1998), respectively (Fig. 4A).The above results indicate that suPAR promotes the activation or expression of apoptosis-related factors and proteins in HK-2 cells and increases the apoptosis susceptibility of cells. Therefore, we attempted to further explore the signaling pathways through which suPAR impacts renal tubular epithelial cells.

After stimulating HK-2 cells with suPAR(24 h), we observed the dilation of the endoplasmic reticulum lumen within the cells by electron microscopy (Fig. 4B). Furthermore, we administered suPAR recombinant protein to C57BL/6 mice via intraperitoneal injection and collected serum and kidney samples 24 h post-injection. While the serum SCr and BUN levels showed mild increases, electron microscopy of the renal cortex revealed endoplasmic reticulum dilation in renal tubular epithelial cells (Supplementary Fig. 3 A, B).

To further confirm whether suPAR promotes ER stress in HK-2 cells, we exposed cells to suPAR for different durations (6 h, 12 h, 24 h). We found that the ER stress sensor protein kinase RNA-like endoplasmic reticulum kinase (PERK) underwent phosphorylation. Subsequently, its downstream factors, eukaryotic initiation factor 2α (EIF2α) and activating transcription factor 4 (ATF4), were activated over time. Meanwhile, the sustained activation of ATF4 promoted the expression of the pro-apoptotic protein C/EBP homologous protein (CHOP). These results demonstrate that suPAR activates PERK-EIF2α-ATF4-CHOP pathway (Fig. 4C). We also observed upregulated PERK-EIF2α-ATF4-CHOP pathway proteins in the renal cortex of C57BL/6 mice after intraperitoneal suPAR injection (Supplementary Fig. 3 C). It is one of the classic pathways of endoplasmic reticulum (ER) stress, and CHOP has been found to mediate cell apoptosis (Sano and Reed 2013). Besides, the protein levels of Nuclear factor erythroid 2-related factor 2 (Nrf2) and Heme oxygenase-1(HO-1) were also increased upon stimulation by suPAR. They are known as regulators of the cellular redox balance and protective antioxidants (Kansanen et al. 2013). However, the expression of HO-1 began to decline at 24 h (Fig. 4C). Concurrently with enhanced EIF2α phosphorylation at the protein level, the EIF2α transcriptional level showed a gradual increase (Fig. 4D).

Following the observation of the fluctuations in the levels of oxidative stress proteins, we next evaluated intracellular ROS level, using DCFH—DA fluorescent probes. The results indicated that the intracellular ROS level did not change significantly when the cells were stimulated with suPAR for 6 h or 12 h, but the ROS level increased after 24—hour stimulation (Fig. 4E). Therefore, we speculate that continuous stimulation of suPAR increases ER stress and oxidative stress in renal tubular epithelial cells and also increases the susceptibility of the cells to apoptosis.

### suPAR binds to RAGE for signal transduction

**Fig. 5:**
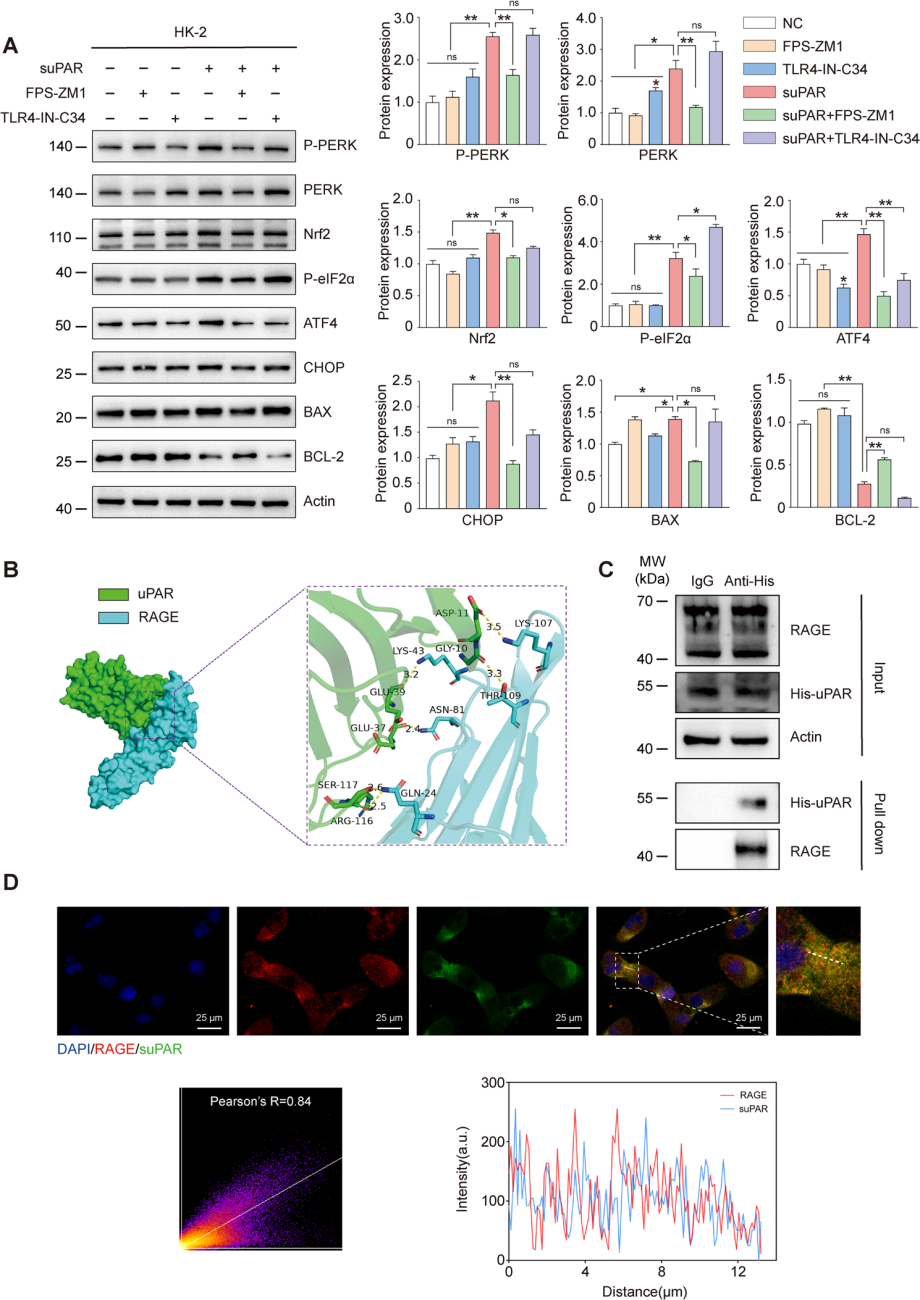
suPAR mediates signal transduction through RAGE binding. **A** Protein levels of P-PERK, PERK, P-eIF2α, ATF4, CHOP, Nrf2, BAX and BCL-2 of HK-2 cells treated with suPAR(10 ng/ml) and/or FPS—ZM1(100 nM) or TLR4—IN—C34(10 μM). **B** The schematic diagram of the Protein docking between uPAR and RAGE. The ARG-116, SER-117, GLU-37, GLU-39, GLY-10, and ASP-11 of uPAR form hydrogen bonds with the GLN-24, ASN-81, GLY-10, THR-109, and LYS-107 of RAGE respectively. The lengths of the hydrogen bonds are 2.5 Å, 2.6 Å, 2.4 Å, 3.2 Å, 3.3 Å, and 3.5 Å respectively. **C** Direct suPAR-RAGE interaction confirmed by pull-down assay coupled with western blotting. **D** Representative confocal images showed the colocalization of suPAR (green) and RAGE (red) in HK-2 cells. Scale bar: 25 μm. Data were presented as mean ± SEM from three independent experiments (*n* = 3). Statistical significance was determined by Kruskal–Wallis test with Dunn's post hoc analysis, with P < 0.05 considered significant (*P < 0.05, **P < 0.01)

Next, we attempted to investigate whether suPAR exerted its active function via receptors on the cell membrane and previous studies have shown that suPAR plays a role in signal transduction through receptors such as RAGE, integrins, and TLR4 (Kim and Dryer 2021; Huang et al. 2023). Therefore, we attempted to explore which receptors are involved in the process of suPAR—promoted ER stress in SA-AKI. RAGE inhibitor FPS—ZM1, TLR4 inhibitor TLR4—IN—C34 and integrins inhibitor CWHM-12 were used to investigate the antagonistic effect. It was observed that RAGE receptor inhibition suppressed suPAR-mediated ER stress and mitochondrial apoptotic protein alterations in HK-2 cells, in contrast to TLR4 or integrin blockade, which had minimal impact. Under suPAR stimulation, the RAGE inhibitor not only reduced the activation of the PERK-EIF2α-ATF4-CHOP pathway and the expression of BAX but also partially rescued the reduction of BCL-2 protein. The TLR4 inhibitor markedly attenuated suPAR-induced ATF4 expression, but showed minimal restorative effects on other proteins (Fig. 5A). Integrin inhibition partially reversed suPAR-mediated elevation of p-PERK and reduction of BCL-2, however, its restorative impact on other proteins remained non-significant (Supplementary Fig. 4). Subsequently, HDOCK and Pymol were used to analyze the interaction between uPAR protein and RAGE. The results showed that the binding energy of RAGE and uPAR is—250.3 kcal/mol, and the residues around the protein—protein interaction interface can form hydrogen bonds to help stabilize the protein—protein complex (Fig. 5B). Coimmunoprecipitation was used to verify that the RAGE protein in the cell lysate can be pulled down by the His—tagged recombinant suPAR protein (Fig. 5C). Immunofluorescence confocal images further showed that FITC—suPAR and the RAGE receptor on the cell surface have significant colocalization (Pearson's R = 0.84) (Fig. 5D). Collectively, these findings implicate RAGE in suPAR signal transduction and demonstrate its contribution to suPAR-triggered endoplasmic reticulum stress and apoptotic protein regulation.

### uPAR monoclonal antibody alleviates acute kidney injury in septic mice

**Fig. 6:**
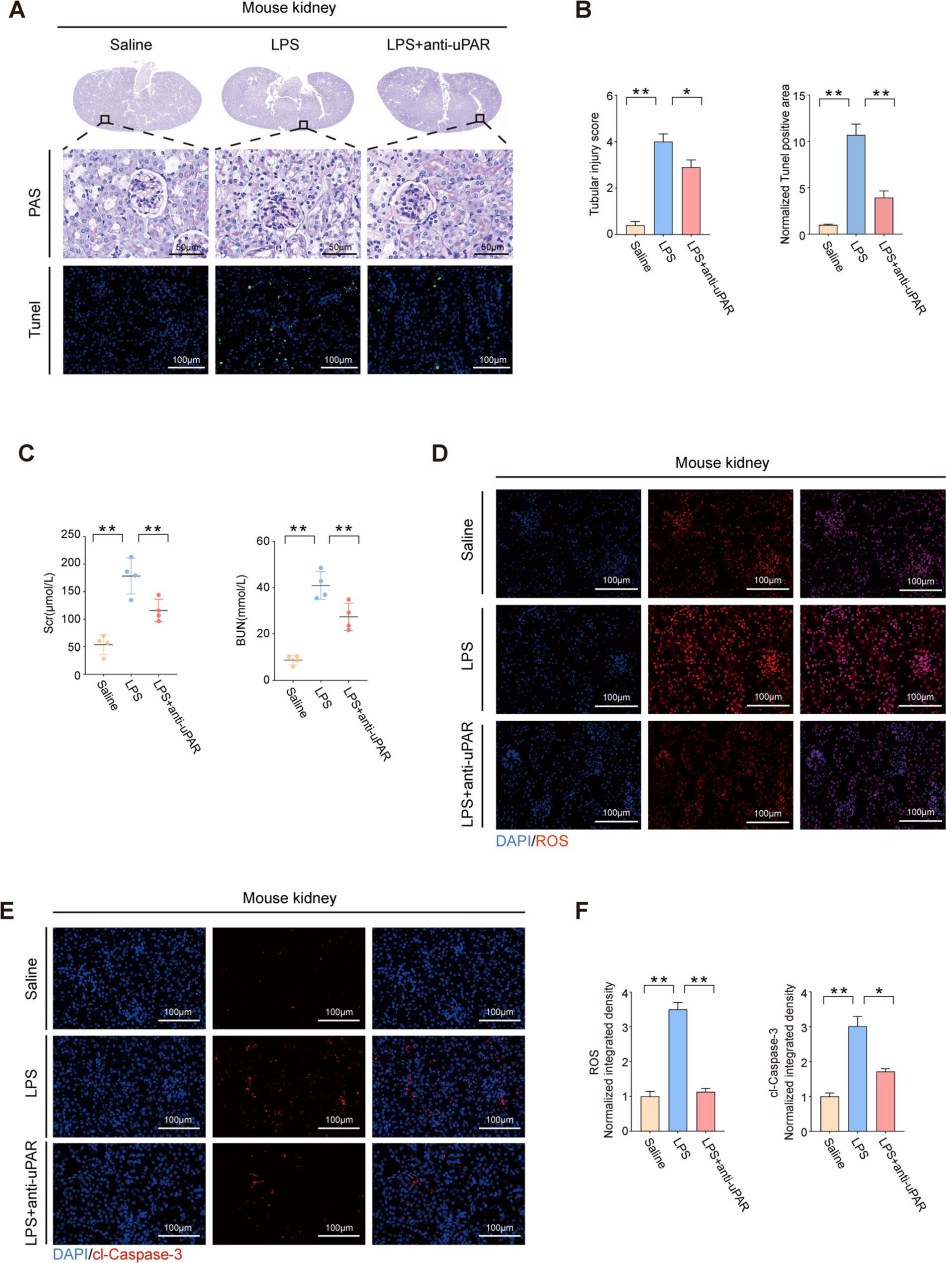
Pretreatment with uPAR monoclonal antibody alleviates acute kidney injury in septic mice. **A**, **B** Representative images of PAS staining, TUNEL staining in kidney tissues of mice injected with Saline(100 μl), LPS (10 mg/kg) for 48 h (with or without pretreatment with 500 μg/kg uPAR monoclonal antibody), along with corresponding quantitative analyses (*n* = 3 mice/group; 3–10 fields/mouse). **C** Serum levels of SCr and BUN of mice in different groups (*n *= 4 mice/group). **D-F** ROS staining of mouse kidney frozen sections, cleaved Caspase-3 (cl-Caspase-3) immunofluorescence staining across different groups, and corresponding quantitative analyses were performed (*n* = 3 mice/group; 3 fields/mouse). Data were presented as mean ± SEM. Statistical significance was determined by one-way ANOVA, with *P* < 0.05 considered significant (*P < 0.05, **P < 0.01)

**Fig. 7:**
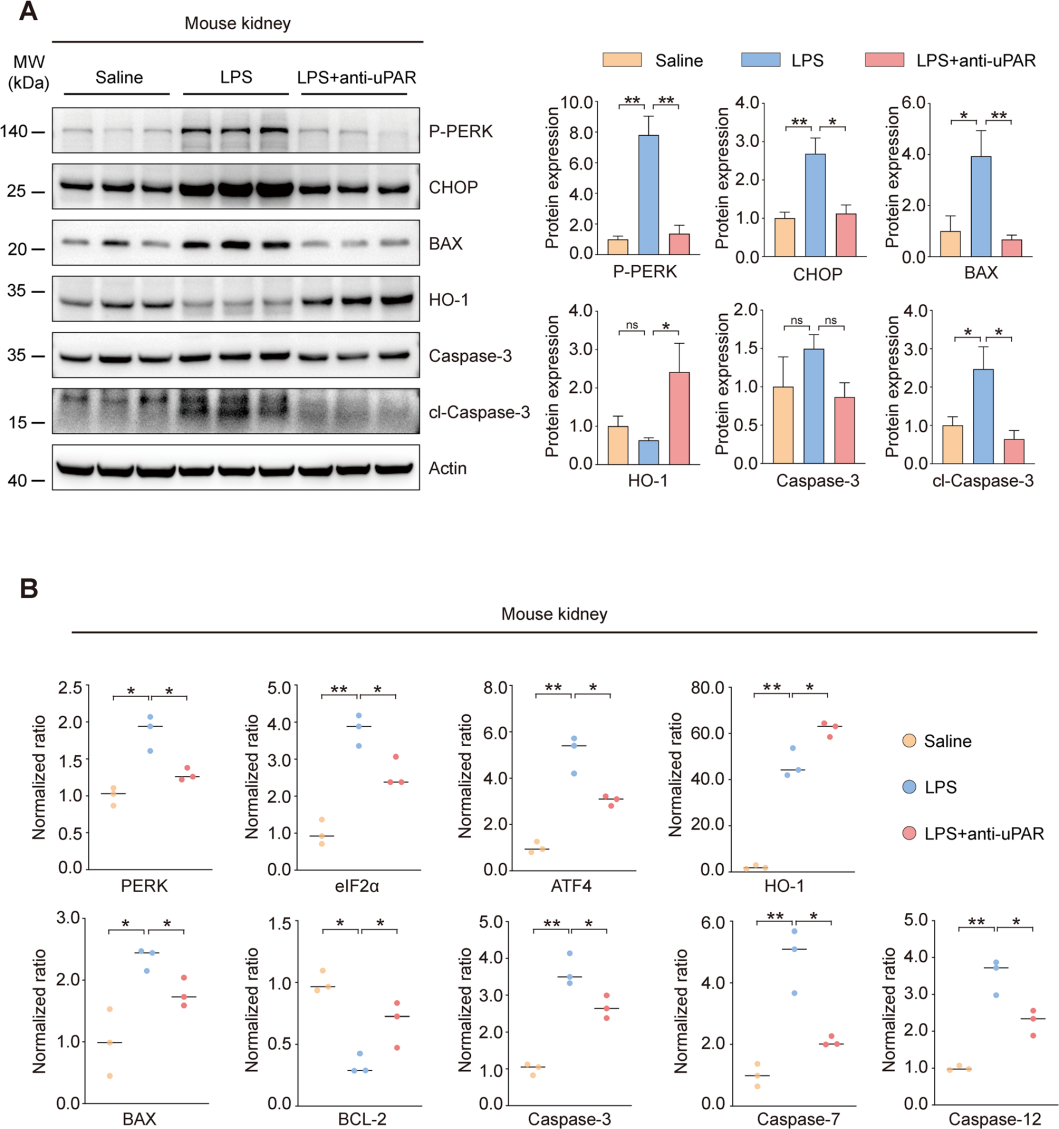
Pretreatment with uPAR monoclonal antibody alleviates ER stress and apoptosis in kidneys of septic mice. **A** Protein levels of P-PERK, CHOP, HO-1, BAX, Caspase3, and cleaved Caspase-3 (cl-Caspase-3) in kidney tissues from mice treated with saline (100 μL), LPS (10 mg/kg) for 48 h, or LPS with uPAR monoclonal antibody pretreatment (500 μg/kg), along with quantitative analysis. **B** mRNA levels of PERK, eIF2α, ATF4, HO-1, BAX, BCL-2, Caspase3, Caspase7 and Caspase12 in kidney tissues across treatment groups (normalized to β-actin). Data were presented as mean ± SEM from three biologically independent mice (*n *= 3). Statistical significance was determined by Kruskal–Wallis test with Dunn's post hoc analysis, with *P* < 0.05 considered significant (*P < 0.05, **P < 0.01)

Based on the above results that high levels of suPAR have negative effects on cells, we further did research on whether antagonizing suPAR could alleviate acute kidney injury in mice. We pretreated the mice with the uPAR monoclonal antibody and then intraperitoneally injected LPS for 48 h for modeling. The results demonstrated that mice pretreated with uPAR monoclonal antibody exhibited attenuated histopathological manifestations, reduced apoptotic cells, and lowered serum SCr and BUN levels compared to SA-AKI model mice (Fig. 6A, B, C). We also observed significantly reduced levels of reactive oxygen species (ROS) and cleaved caspase-3 (cl-caspase-3) in the renal cortex of monoclonal antibody-pretreated mice (Fig. 6D, E, F). Simultaneously, through western blot and qPCR, we confirmed that pretreatment with uPAR monoclonal antibody alleviated ER stress and apoptosis in the renal cortex of SA—AKI mice. The decrease in cleaved—caspase 3 was accompanied by a substantial increase in the anti—apoptotic protein HO-1 (Fig. 7A, B) (Wegiel et al. 2014; Ryter 2021). These results indicated that neutralizing suPAR/uPAR could alleviate kidney injury in SA—AKI mice by inhibiting ER stress and apoptosis.

## Discussion

Our study findings are consistent with those of some other clinical studies and have provided some evidence supporting the potential of suPAR in predicting AKI (Hayek et al. 2020; Nusshag et al. 2023; Azam et al. 2020). But it should be acknowledged that our study was constrained by a relatively modest clinical sample size, precluding subgroup analyses and adjustment for potential confounding factors. This hindered exploration of differences in suPAR's discriminative performance across clinically distinct subgroups (e.g., age strata, sex, AKI etiology, and comorbidities). Consequently, the generalizability of our findings to specific patient populations remains uncertain. Moreover, inadequately adjusted factors could potentially bias the observed association between suPAR and AKI. Notably, the optimal cutoff value determined in our study was 4.28 ng/ml, which closely approximated the cutoff value (4.26 ng/mL) reported by Anne et al. in their research on acute kidney injury among elderly (≥ 65 years old) medical inpatients (Walls et al. 2021). By contrast, in patients undergoing percutaneous coronary intervention (PCI) and sepsis patients, the optimal cutoff values were established as 3.305 ng/mL and 6.31 ng/mL, respectively (Qin et al. 2021; Zhang et al. 2024). These findings further underscore the necessity of customizing suPAR threshold values according to patient demographic characteristics and AKI etiologies. Thus, large-scale, multi-center prospective studies are warranted to address the generalizability of suPAR as an AKI biomarker and to formulate adjustment strategies for suPAR thresholds across diverse populations.

Despite suPAR's predictive association with AKI in our cohort, its established role as a systemic inflammation biomarker raises legitimate specificity concerns. suPAR is recognized by many researchers as a biomarker for chronic kidney disease, chronic systemic inflammation, cardiovascular disease, and malignant tumors (Rasmussen et al. 2021; Hayek et al. 2015; Goodchild et al. 2022; Hindy et al. 2022; Liu et al. 2017). In addition, human suPAR levels are determined by a variety of factors, including inheritance, lifestyle, and acute and chronic diseases. The contribution of these factors to suPAR has not yet been fully elucidated, and suPAR may represent the overall effect of these factors (Rasmussen et al. 2021). Among the ICU patients we included, there was also a case of severe pneumonia in which AKI never occurred while suPAR remained at a significantly high level. Specifically, the serum suPAR concentrations during the five—day period starting from the enrollment were 12.50 ng/ml, 10.61 ng/ml, 13.21 ng/ml, 10.58 ng/ml, and 12.60 ng/ml respectively. Such instances underscore that suPAR elevation, while indicative of inflammatory burden, may not invariably translate to end-organ damage, reflecting its inherent limitation as a standalone AKI-specific biomarker. suPAR’s performance contrasts with tissue-injury-focused biomarkers like urinary [TIMP-2]•[IGFBP7] (specific to renal tubular stress) or NGAL (reflecting neutrophil activation in direct renal injury), which exhibit higher organ specificity but lack suPAR’s capacity to capture upstream systemic risk (Ostermann et al. 2020). Thus, future studies can focus on the combined diagnostic efficacy of suPAR with other AKI biomarkers, integrating suPAR’s sensitivity to inflammatory priming with damage-effect biomarkers.

Previous studies have found that renal tubular epithelial cells exposed to suPAR have a higher energy demand, accompanied by an increase in mitochondrial basal respiration and ATP production (Hayek et al. 2020). Therefore, our study focused on the relationship between suPAR and oxidative stress and confirmed that suPAR promotes ER stress and increases the sensitivity to apoptosis. ER stress, as a protective stress response of cells, can reduce the concentration of unfolded proteins in cells, but persistent and severe ER stress leads to programmed cell death (Sano and Reed 2013; Zhang et al. 2022). Consistent with the research on podocytes by Kim et al. (Kim and Dryer 2024), We found that the intracellular ROS level of HK-2 cells was not significantly changed when stimulated by suPAR for less than 24 h, while it increased upon continuous stimulation for 24 h. We also observed that, in HK-2 cells, suPAR upregulated NRF2/HO1 first, and the HO-1 protein level started to drop at 24 h. Moreover, in the mouse renal cortex, antagonizing suPAR significantly elevated the HO-1 level. Based on the above results, we believe that circulating suPAR may be involved in the regulation of the oxidative stress system in renal tubular epithelial cells. In the early stage, it promotes the activation of ER stress and the antioxidant system, helping cells survive during stress responses (Sano and Reed 2013; Loboda et al. 2016). However, renal tubular epithelial cells that are continuously stimulated by suPAR will face oxidative stress imbalance and programmed cell death. Accordingly, the persistently high level of serum suPAR in patients has certain guiding significance for taking more active treatment measures in clinical practice. Additionally, although our preliminary in vivo experiments demonstrate protective effects of anti-uPAR therapy against AKI, further elucidation of its underlying pathophysiological mechanisms and optimization of monoclonal antibody administration parameters (dose, timing, delivery method) are required.

We have confirmed that suPAR can also bind to RAGE, promoting cellular oxidative stress responses and activating inflammatory factors. RAGE is a multi-ligand receptor and a member of the immunoglobulin (Ig) superfamily (Fritz 2011). The interaction between cell-surface RAGE and its ligands participates in cell cascades that lead to inflammatory phenotypes both in vitro and in vivo (Dong et al. 2022). Under physiological conditions, the expression level of RAGE is relatively low (Otazu et al. 2021). However, in chronic and persistent inflammatory conditions, RAGE signal transduction is upregulated and involved in the development of various diseases (Dong et al. 2022). Therefore, for populations with chronically elevated serum suPAR levels, it is worthy of further investigation whether suPAR can increase the expression of cell-surface RAGE, thus aggravating their susceptibility to various diseases including AKI.

There are still several significant limitations in this study. First, our observational studies encompassed a relatively small number of patients, which was insufficient for subgroup analysis or adjusting for more confounding factors, thereby potentially leading to biased results. Besides, the lack of longitudinal suPAR measurements beyond enrollment limits the understanding of its dynamic changes in AKI progression. Second, constrained by project funding, suPAR—transgenic mice were not utilized to strengthen the research on the suPAR mechanism in AKI mice. Additionally, the synergistic or perhaps antagonistic effects of suPAR and LPS were not successfully verified in vitro experiments, since there was evidence indicating that both could bind to TLR4 for signal transduction (Huang et al. 2023; Park et al. 2009; Ciesielska et al. 2021). Moreover, we have only explored the role of suPAR in apoptosis and have not delved into other equally crucial cell death pathways in acute kidney injury. Nevertheless, we still believe that these shortcomings will be overcome in future studies.

## Conclusions

**Fig. 8:**
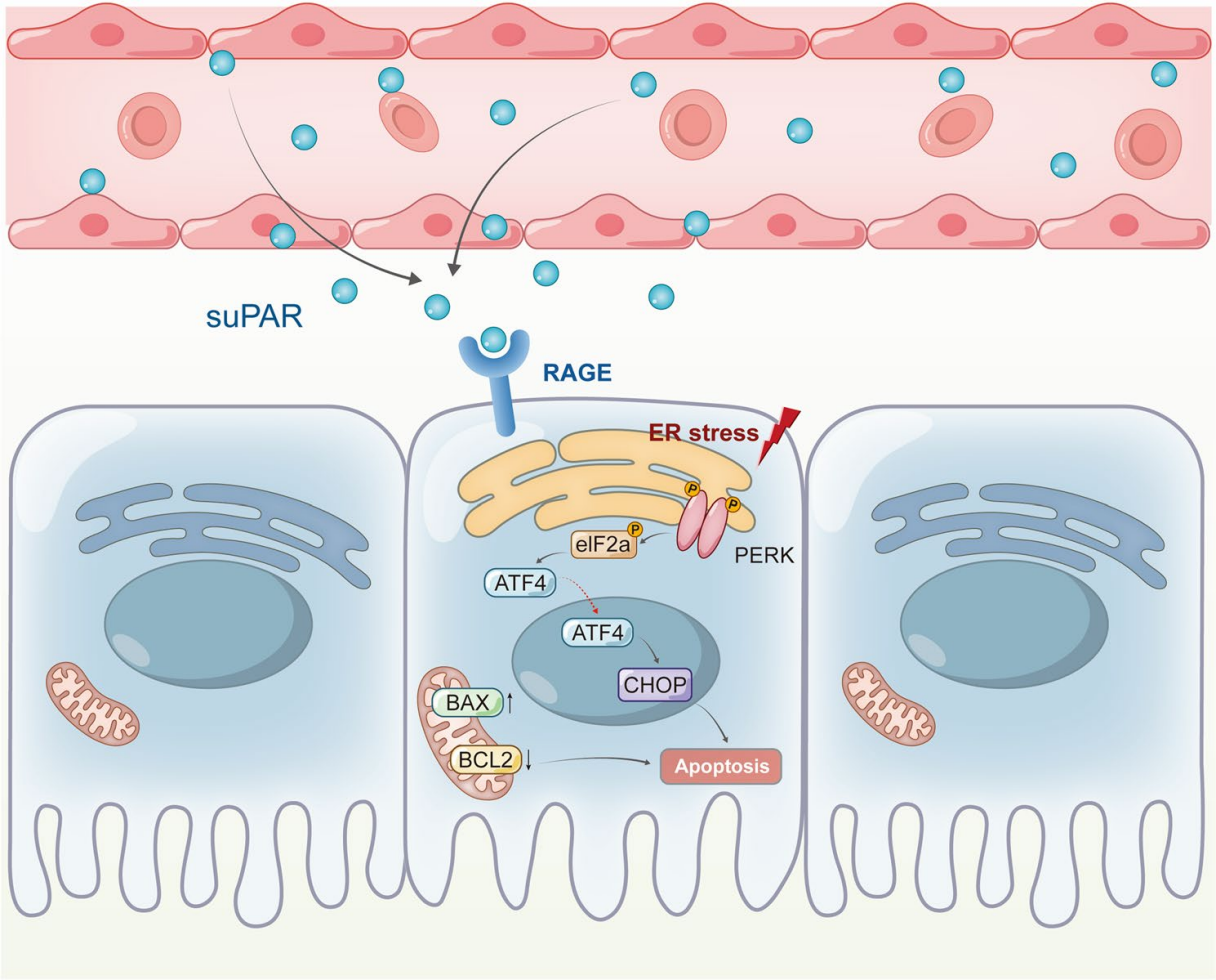
Schematic summary of our study

Our clinical data suggest that high levels of suPAR are associated with acute kidney injury in our patients. We also demonstrated that the persistently elevated circulating suPAR activates the PERK-EIF2α-ATF4-CHOP pathway and promotes the expression of apoptosis—related proteins in renal tubular epithelial cells, and that RAGE plays an important role in the signal transduction of suPAR (Fig. 8). Neutralizing suPAR/uPAR may provide new therapeutic potential for kidney injury.

## Supplementary Information

Supplementary Material 1

Supplementary Material 2

Supplementary Material 3. Fig. S1 ROC curve analysis of suPAR, CysC, and β2-MG for AKI prediction in ICU patients. A. The ROC analysis compares the diagnostic performance of suPAR, CysC, and β2-MGin discriminating AKIfrom non-AKIpatients in an ICU validation cohort

Supplementary Material 4. Fig. S2 Elevated suPAR/uPAR expression in kidney tissues of septic AKI and cytokine storm-induced AKI mouse models. A. Serum levels of SCr and BUN in mice injected with Saline, LPSfor 48 h, TNF - αand IFN - γfor 6 h. B-C. Representative images of PAS staining, TUNEL staining, and uPAR immunohistochemical staining in kidney tissues across experimental groups, along with quantitative analyses. D-E. uPAR immunofluorescence staining and quantitative assessment were performed in different groups of mice, with LTL staining used to visualize the brush border of renal tubular epithelial cells. F. Protein expression levels of suPAR/uPAR in kidney tissues from different groups. G. Protein expression levels of suPAR/uPAR in kidney tissues from mice injected with LPSat different time points. Data were presented as mean ± SEM. Statistical significance was determined by one-way ANOVA unless noted, with P < 0.05 considered significant

Supplementary Material 5. Fig. S3 Recombinant suPAR administration induces endoplasmic reticulum dilation and ER stress in renal tubular epithelial cells. A. Serum levels of SCr and BUN in mice injected with Salineor recombinant suPARfor 24 h. B. Representative images of transmission electron microscope for endoplasmic reticulum in tubular epithelial cells of mice. Scale bar: 0.5μm. C. Protein levels of P-PERK, PERK, P-eIF2α, ATF4, CHOP in kidney tissues from different groups. Data were presented as mean ± SEM. Statistical significance was determined by Mann-Whitney U test, with P < 0.05 considered significant

Supplementary Material 6. Fig. S4 Limited restorative effects of integrin blockade on suPAR-driven ER stress signaling and apoptosis-related protein expression. A. Protein levels of P-PERK, PERK, P-eIF2α, ATF4, CHOP, Nrf2, BAX and BCL-2 of HK-2 cells treated with suPARand/or CWHM-12, along with quantitative analyses. Data were presented as mean ± SEM from three independent experiments. Statistical significance was determined by one-way ANOVA, with P < 0.05 considered significant

## Data Availability

All data generated or analyzed during this study are included in this published article and its supplementary information files.

## Abbreviations

AKI: Acute kidney injury
suPAR: Soluble urokinase plasminogen activator receptor
ICU: Intensive care unit
ER stress: Endoplasmic reticulum stress
ROS: Reactive oxygen species
RAGE: The receptor of advanced glycation end products
CKD: Chronic kidney disease
SCr: Serum creatinine
TIMP-2: Tissue me talloproteinase-2
IGFBP7: Insulin-like growth factor binding protein 7
KIM-1: Kidney injury molecule 1
NGAL: Neutrophil gelatinase–associated lipocalin
uPAR: Urokinase plasminogen activator receptor
uPA: Urokinase plasminogen activator
TLR4: Toll-like receptor 4
FBS: Fetal bovine serum
BUN: Blood urea nitrogen
PAS: Periodic acid-Schiff staining
RIPA: Samples containing radioimmunoprecipitation assay
TEM: Transmission electron microscope
ANOVA: One-way analysis of variance
L.S.D: Least squares difference
NF-κB: Nuclear Factor Kappa-light-chain-enhancer of Activated B Cells
BAX: Bcl-2-Associated X Protein
BCL-2: B-cell lymphoma/leukemia-2
PERK: Protein kinase RNA-like endoplasmic reticulum kinase
EIF2α: Eukaryotic initiation factor 2α
ATF4: Activating transcription factor 4
CHOP: C/EBP homologous protein
Nrf2: Nuclear factor erythroid 2-related factor 2
HO-1: Heme oxygenase-1
LTL: Lotus tetragonolobus lectin
Cys-C: Cystatin C
β2-MG: β2-Microglobulin
LOOCV: Leave-one-out cross-validation
ROC: Receiver operating characteristic
