# Supplementary material for: Soluble urokinase plasminogen activator receptor promotes endoplasmic reticulum stress and apoptosis susceptibility through RAGE in sepsis acute kidney injury
Source: Mol Med. 2025 Sep 26;31:296. doi: 10.1186/s10020-025-01352-w (PMC12465643; doi:10.1186/s10020-025-01352-w)
Supplement: Supplementary file 1 — Supplementary Material 1 [file 10020_2025_1352_MOESM1_ESM.docx]

**Supplementary Table 1**—**Demographic and Clinical Characteristics of Enrolled Patients^§^**

| **Characteristic** | **No**  **Acute Kidney Injury(N=33)** | **AKI occurred after enrollment(N=17)** | **P Value** |
| --- | --- | --- | --- |
| Demographics |  |  |  |
| Age — yr | 61.2±14.8 | 65.2±11.1 | 0.287 |
| Male sex — no. (%)  Body-mass index, kg/m2  Comorbidities, n/*N* (%) | 19(57.6)  23.7±41 | 11(64.7)  23.3±5.5 | 0.626  0.946 |
| Chronic kidney disease | 2(6.1) | 2(11.8) | 0.481 |
| Type 2 diabetes mellitus  COPD*  Heart failure  Chronic liver disease  Hypertension  Anaemia  Primary diagnosis for ICU^#^ admission, n (%) | 7 (21.2)  0 (0.0)  2 (6.1)  1 (3.0)  13(39.4)  0 (0.0) | 4(23.5)  3(17.6)  1(5.9)  3(17.6)  10(58.8)  3(17.6) | 0.851  0.013  0.980  0.071  0.192  0.013 |
| Neurologic  Respiratory  Cardiovascular  Trauma  Gastrointestinal  Shock  Sepsis  Relevant examinations at enrollment | 2(6.1)  5(15.2)  5(15.2)  8(24.2)  1(3.0)  10(30.3)  11(33.3) | 0(0.0)  1(5.9)  4(23.5)  0(0.0)  1(5.9)  5(29.4)  5(29.4) | 0.300  0.339  0.465  0.027  0.626  0.948  0.778 |
| Median serum creatinine (IQR)—μmol/L  Median suPAR^ǂ^ level (IQR)—ng/ml  Median serum cystatin C (IQR)—mg/L  Median serum β2-microglobulin (IQR)—mg/L | 80.1(59.0-103.0)  2.9(1.1-3.4)  1.2(0.7-1.6)  2.1(1.3-2.8) | 115.2(76.0-121.0)  5.2(2.4-7.1)  1.9(1.3-2.3)  3.7(2.7-4.4) | 0.046  0.006  0.004  <0.001 |

**^§^**Plus–minus values are means±SD , IQR denotes interquartile range.

*Chronic obstructive pulmonary disease (COPD)

^#^ Intensive care unit (ICU)

^ǂ^ Soluble urokinase plasminogen activator receptor(suPAR)
