# Supplementary material for: Soluble urokinase plasminogen activator receptor promotes endoplasmic reticulum stress and apoptosis susceptibility through RAGE in sepsis acute kidney injury
Source: Mol Med. 2025 Sep 26;31:296. doi: 10.1186/s10020-025-01352-w (PMC12465643; doi:10.1186/s10020-025-01352-w)
Supplement: Supplementary file 2 — Supplementary Material 2 [file 10020_2025_1352_MOESM2_ESM.docx]

**Supplementary Table 2 Primer Sequences**

| Gene | Forward | Reverse |
| --- | --- | --- |
| eIF2α (human) | CTGGACCTCATGCAGCTTTAGC | CTCCATAGTAGGAAGCTCCTGTC |
| Actin (human) | CACCATTGGCAATGAGCGGTTC | AGGTCTTTGCGGATGTCCACGT |
| PERK (mouse) | CCGATGTCAGTGACAACAGCTG | AAGACAACGCCAAAGCCACCAC |
| eIF2α (mouse) | CCACACTTCACAGAAAGCACGG | TCGAAGGAGTGCAGTAGTCCCT |
| ATF4 (mouse) | AACCTCATGGGTTCTCCAGCGA | CTCCAACATCCAATCTGTCCCG |
| HO-1 (mouse) | CACTCTGGAGATGACACCTGAG | GTGTTCCTCTGTCAGCATCACC |
| BAX (mouse) | AGGATGCGTCCACCAAGAAGCT | TCCGTGTCCACGTCAGCAATCA |
| BCL-2 (mouse) | CCTGTGGATGACTGAGTACCTG | AGCCAGGAGAAATCAAACAGAGG |
| Caspase-3 (mouse) | GGAGTCTGACTGGAAAGCCGAA | CTTCTGGCAAGCCATCTCCTCA |
| Caspase-7 (mouse) | CCGTCCACAATGACTGCTCTTG | CCCGTAAATCAGGTCCTCTTCC |
| Caspase-12 (mouse) | CAGATGAGGAACGTGTGTTGAGC | GGAACCAGTCTTGCCTACCTTC |
| Actin (mouse) | CATTGCTGACAGGATGCAGAAGG | TGCTGGAAGGTGGACAGTGAGG |
| uPAR (mouse) | AGGACTACCGTGCTTCGGGAAT | ACACGGTCTCTGTCAGGCTGAT |
