# Supplementary figures and images for: Soluble urokinase plasminogen activator receptor promotes endoplasmic reticulum stress and apoptosis susceptibility through RAGE in sepsis acute kidney injury
Source: Mol Med. 2025 Sep 26;31:296. doi: 10.1186/s10020-025-01352-w (PMC12465643; doi:10.1186/s10020-025-01352-w)

Supplementary Figure 1

A

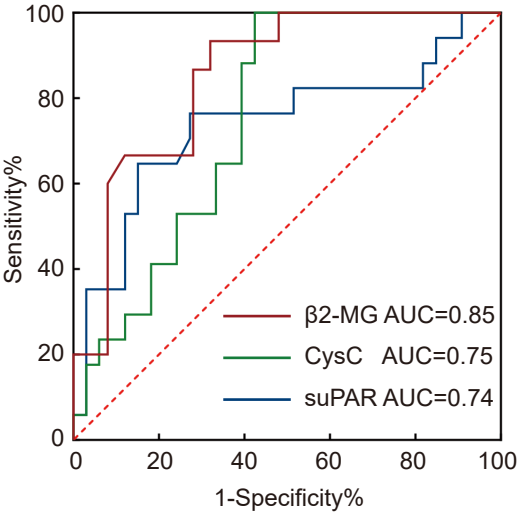

Supplement: Supplementary file 3 — Supplementary Material 3. Fig. S1 ROC curve analysis of suPAR, CysC, and β2-MG for AKI prediction in ICU patients. A. The ROC analysis compares the diagnostic performance of suPAR, CysC, and β2-MGin discriminating AKIfrom non-AKIpatients in an ICU validation cohort [file 10020_2025_1352_MOESM3_ESM.pdf]

Supplementary Figure 2

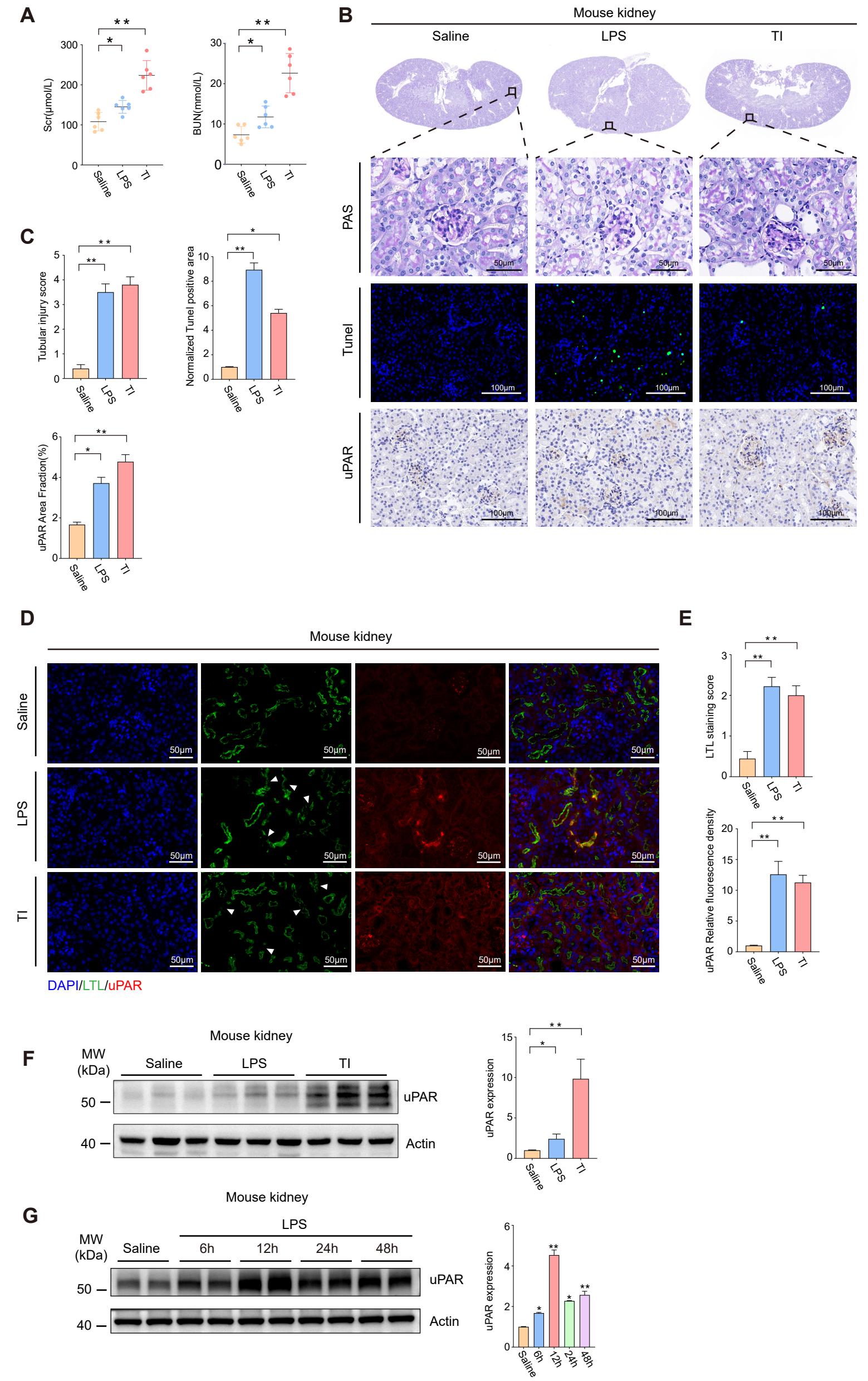

Supplement: Supplementary file 4 — Supplementary Material 4. Fig. S2 Elevated suPAR/uPAR expression in kidney tissues of septic AKI and cytokine storm-induced AKI mouse models. A. Serum levels of SCr and BUN in mice injected with Saline, LPSfor 48 h, TNF - αand IFN - γfor 6 h. B-C. Representative images of PAS staining, TUNEL staining, and uPAR immunohistochemical staining in kidney tissues across experimental groups, along with quantitative analyses. D-E. uPAR immunofluorescence staining and quantitative assessment were performed in different groups of mice, with LTL staining used to visualize the brush border of renal tubular epithelial cells. F. Protein expression levels of suPAR/uPAR in kidney tissues from different groups. G. Protein expression levels of suPAR/uPAR in kidney tissues from mice injected with LPSat different time points. Data were presented as mean ± SEM. Statistical significance was determined by one-way ANOVA unless noted, with P < 0.05 considered significant [file 10020_2025_1352_MOESM4_ESM.pdf]

# Supplementary Figure 3

**A**

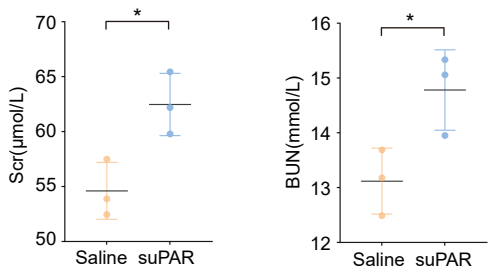

**B**

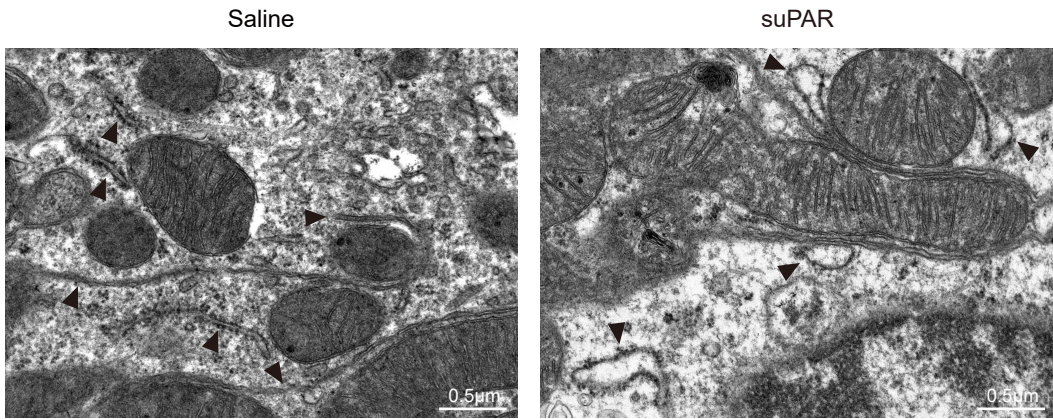

**C**

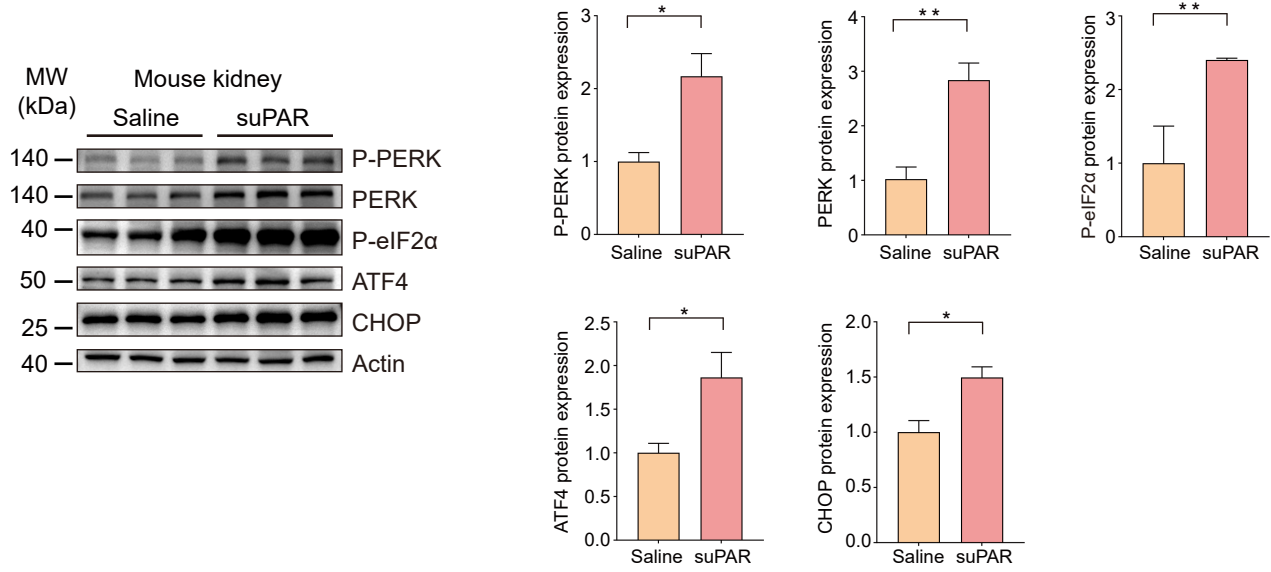

Supplement: Supplementary file 5 — Supplementary Material 5. Fig. S3 Recombinant suPAR administration induces endoplasmic reticulum dilation and ER stress in renal tubular epithelial cells. A. Serum levels of SCr and BUN in mice injected with Salineor recombinant suPARfor 24 h. B. Representative images of transmission electron microscope for endoplasmic reticulum in tubular epithelial cells of mice. Scale bar: 0.5μm. C. Protein levels of P-PERK, PERK, P-eIF2α, ATF4, CHOP in kidney tissues from different groups. Data were presented as mean ± SEM. Statistical significance was determined by Mann-Whitney U test, with P < 0.05 considered significant [file 10020_2025_1352_MOESM5_ESM.pdf]

Supplementary Figure 4

A

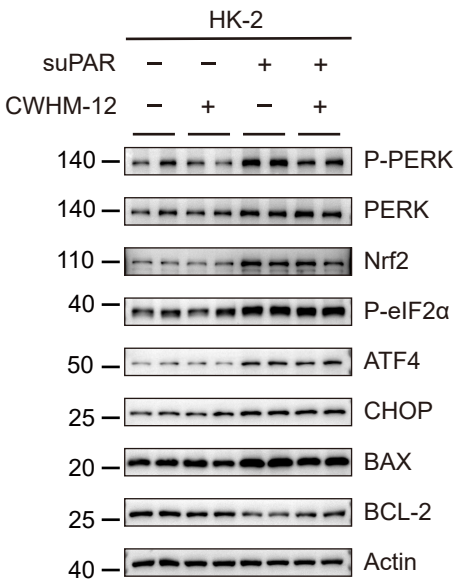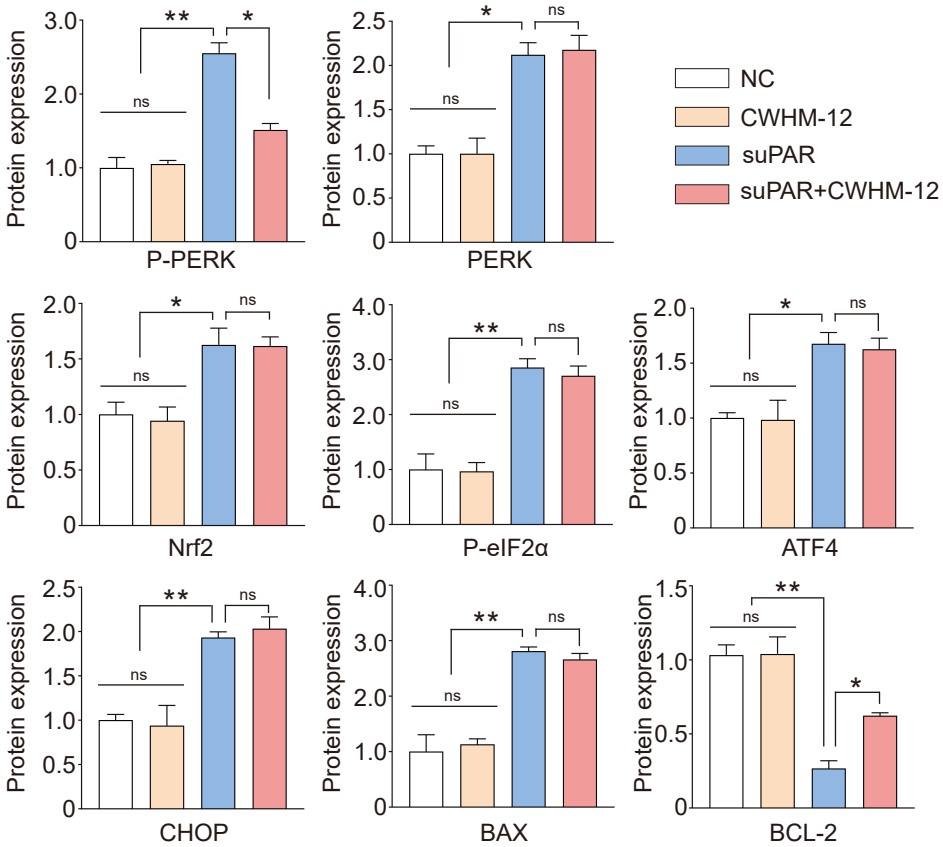

Supplement: Supplementary file 6 — Supplementary Material 6. Fig. S4 Limited restorative effects of integrin blockade on suPAR-driven ER stress signaling and apoptosis-related protein expression. A. Protein levels of P-PERK, PERK, P-eIF2α, ATF4, CHOP, Nrf2, BAX and BCL-2 of HK-2 cells treated with suPARand/or CWHM-12, along with quantitative analyses. Data were presented as mean ± SEM from three independent experiments. Statistical significance was determined by one-way ANOVA, with P < 0.05 considered significant [file 10020_2025_1352_MOESM6_ESM.pdf]
